# Optical Quantal Analysis

**DOI:** 10.3389/fnsyn.2019.00008

**Published:** 2019-03-26

**Authors:** Matthew J. MacDougall, Alan Fine

**Affiliations:** Department of Physiology and Biophysics, Faculty of Medicine, Dalhousie University, Halifax, NS, Canada

**Keywords:** synaptic plasticity, synaptic potency, synaptic reliability, LTP (long-term potentiation), two photon microscopy

## Abstract

Understanding the mechanisms by which long-term synaptic plasticity is expressed remains an important objective in neuroscience. From a physiological perspective, the strength of a synapse can be considered a consequence of several parameters including the probability that a presynaptic action potential (AP) evokes the release of neurotransmitter, the mean number of quanta of transmitter released when release is evoked, and the mean amplitude of a postsynaptic response to a single quantum. Various methods have been employed to estimate these quantal parameters from electrophysiological recordings; such “quantal analysis” has been used to support competing accounts of mechanisms of expression of long-term plasticity. Because electrophysiological recordings, even with minimal presynaptic stimulation, can reflect responses arising at multiple synaptic sites, these methods are open to alternative interpretations. By combining intracellular electrical recording with optical detection of transmission at individual synapses, however, it is possible to eliminate such ambiguity. Here, we describe methods for such combined optical and electrical monitoring of synaptic transmission in brain slice preparations and illustrate how quantal analyses thereby obtained permit more definitive conclusions about the physiological changes that underlie long-term synaptic plasticity.

## Introduction

Physiological and anatomical characterization of synapses provides ongoing and central challenges to neuroscience. Paramount among these challenges is clarification of the mechanisms that govern activity-dependent changes in synaptic strength, such as long-term potentiation (LTP; Bliss and Lømo, 1973; Bliss and Gardner-Medwin, 1973) and long-term depression (LTD; Dudek and Bear, 1992), the purported cellular basis of learning and memory, continues to be an essential objective. While the *induction* of LTP at CA3-CA1 synapses is generally agreed to be chiefly a postsynaptic phenomenon, controversy remains with respect to the locus and nature of changes responsible for the *expression* of LTP at these synapses (Bliss and Collingridge, 2013; Granger and Nicoll, 2014; MacDougall and Fine, 2014; see Bear and Abraham, 1996; Collingridge et al., 2010 for reviews on LTD). Here, we present a brief summary of advances in the understanding of this issue, followed by a description of optical quantal analysis, a powerful method employed by our laboratory to investigate unitary synaptic function.

### Classical Quantal Analysis

The pioneering work of Fatt and Katz (1952) and Del Castillo and Katz (1954) demonstrated that the release of transmitter substances occur in multi-molecular packets, now known to be synaptic vesicles (Gray, 1959), at the frog neuromuscular junction. According to this model, the smallest electrical response at a synapse results from the release of a single vesicle or quantum of transmitter (Del Castillo and Katz, 1954; Boyd and Martin, 1956). Postsynaptic responses to evoked neurotransmitter release are therefore said to be quantal in nature; i.e., they reflect the summation of a number of discrete events due to the exocytosis of vesicular contents of neurotransmitter. Quantal analysis is a statistical procedure used to isolate the mechanistic components of synaptic transmission and their modifications (Del Castillo and Katz, 1954; Boyd and Martin, 1956). Attempts to assess the role of changes in these components in synaptic plasticity *via* quantal analysis of electrophysiological recordings of CA1 hippocampal synapses before and after induction of plasticity have been inconclusive (Voronin, 1994), with competing accounts supporting pre- (Voronin, 1983; Bekkers and Stevens, 1990; Larkman et al., 1991; Malinow, 1991; Tsien and Malinow, 1991; Voronin et al., 1992), post- (Foster and McNaughton, 1991; Isaac et al., 1996a,b), and in some instances a combination of pre- and postsynaptic components of plasticity expression (Kullmann and Nicoll, 1992; Larkman et al., 1992). All such attempts, however, have been susceptible to alternative interpretations and have been at the center of a continuing “locus debate” in LTP research (Nicoll, 2003; Kerchner and Nicoll, 2008; MacDougall and Fine, 2014). The sources of divergence may include differences in tissue preparation and times of analysis, but criticisms have largely focused on the heterogeneity of central synapses, the uncertain applicability of theoretical assumptions, and the fact that postsynaptic responses, even with minimal presynaptic stimulation, result from an unknown number of activated synapses, all of which complicate conclusions about unitary responses (Redman, 1990; Faber and Korn, 1991; Korn and Faber, 1991; Walmsley, 1995).

### Fluorescence Microscopy and Dendritic Spines

The long-term visualization of individual dendritic spines using confocal fluorescence microscopy before and after LTP (Hosokawa et al., 1995) as well as the visualization of dendritic and spine Ca^2+^ signals (Connor et al., 1994; Malinow et al., 1994; Yuste and Denk, 1995; Emptage et al., 1999; Mainen et al., 1999; Yuste et al., 1999; Kovalchuk et al., 2000; Reid et al., 2001; Sabatini et al., 2002) during synaptic stimulation have greatly influenced the field of synaptic plasticity and have become indispensable techniques used to probe synaptic function. These technological and analytical developments, coupled with the statistical approach of classical quantal analysis, opened the possibility of optical quantal analysis of LTP at individual hippocampal synapses (Emptage et al., 2003).

### Optical Quantal Analysis

Optical quantal analysis combines classical electrophysiological recording with optical monitoring of fluorescent Ca^2+^ indicators in dendritic spines. Optical detection of synaptically-evoked postsynaptic Ca^2+^ transients [EPSCaTs (pronounced epps’kats); Malinow et al., 1994; Yuste and Denk, 1995; Emptage et al., 1999] has given researchers a means to overcome many of the analytical and interpretational difficulties associated with classical quantal analysis. EPSCaTs in CA1 pyramidal cells are triggered by small synaptically-evoked Ca^2+^ influx through NMDA receptors, amplified by Ca^2+^-induced Ca^2+^ release (CICR) from internal stores (Emptage et al., 1999) and display stochastic failures (Yuste and Denk, 1995; Emptage et al., 1999) corresponding to the statistical nature of transmitter release. Postsynaptic EPSCaT detection thus serves as a readout of presynaptic transmitter release from the directly apposed synaptic bouton. Here we review technical aspects of the procedure including simultaneous electrophysiological and optical recording, explain statistical aspects of their conjoint analysis, and illustrate some important conclusions thereby obtained.

## Materials and Methods

### Hippocampal Slices

Transverse 350 μm slices of hippocampus, which retain much of the functional and structural integrity of the original tissue, are cut from 2 to 3-week-old male Wistar rats, according to standard protocols (e.g., Skrede and Westgaard, 1971; Geiger et al., 2002; Bischofberger et al., 2006; see Aitken et al., 1995 for discussion). We dissect hippocampal tissue in ice-cold sucrose-based cutting solution containing (in mM): 105 Sucrose, 50 NaCl, 1.25 NaH_2_PO_4_, 2.5 KCl, 26 NaHCO_3_, 13 Glucose, 0.5 CaCl_2_, 7 MgCl_2_. Dissected hippocampi are then laid out in an agar block perpendicular to the cutting blade, and slices cut perpendicular to the longitudinal axis of the hippocampus using a vibrating tissue slicer (Leica VT1200, Leica Biosystems, Nussloch). Slices are then transferred to a custom interface chamber with supporting mesh and allowed to recover for 30–60 min at 32–33°C while oxygenated with 95% O_2_/5% CO_2_. Under these conditions, the slices remain viable for up 8 h. Alternatively, organotypic hippocampal slice cultures may be cut from 7 to 21 day-old male Wistar rat pups according to published methods (Yamamoto et al., 1989; Stoppini et al., 1991), placed on Millicell CM inserts (Millipore, Bedford, MA, USA) with media replaced every 2–3 days, and maintained for 1–3 weeks *in vitro* prior to recording. For recording, acute slices or organotypic slice cultures on their supporting membranes are transferred to a specially designed chamber where they are continually superfused (~2 ml/min) with oxygenated (95% O_2_/5% CO_2_) artificial cerebrospinal fluid (ACSF) containing (in mM): 120 NaCl, 3 KCl, 1 MgCl_2_, 2–3 CaCl_2_, 1.2 NaH_2_PO_4_, 23 NaHCO_3_, 11 glucose. ACSF should be maintained at near physiological temperatures (32–33°C) using a temperature control unit throughout the duration of experiments. Both methods of tissue preparation have been shown to yield similar physiological synaptic properties, with organotypic slices displaying greater connectivity (De Simoni et al., 2003), including aberrant recurrent connections.

### Microscopy

**Figure 1:**
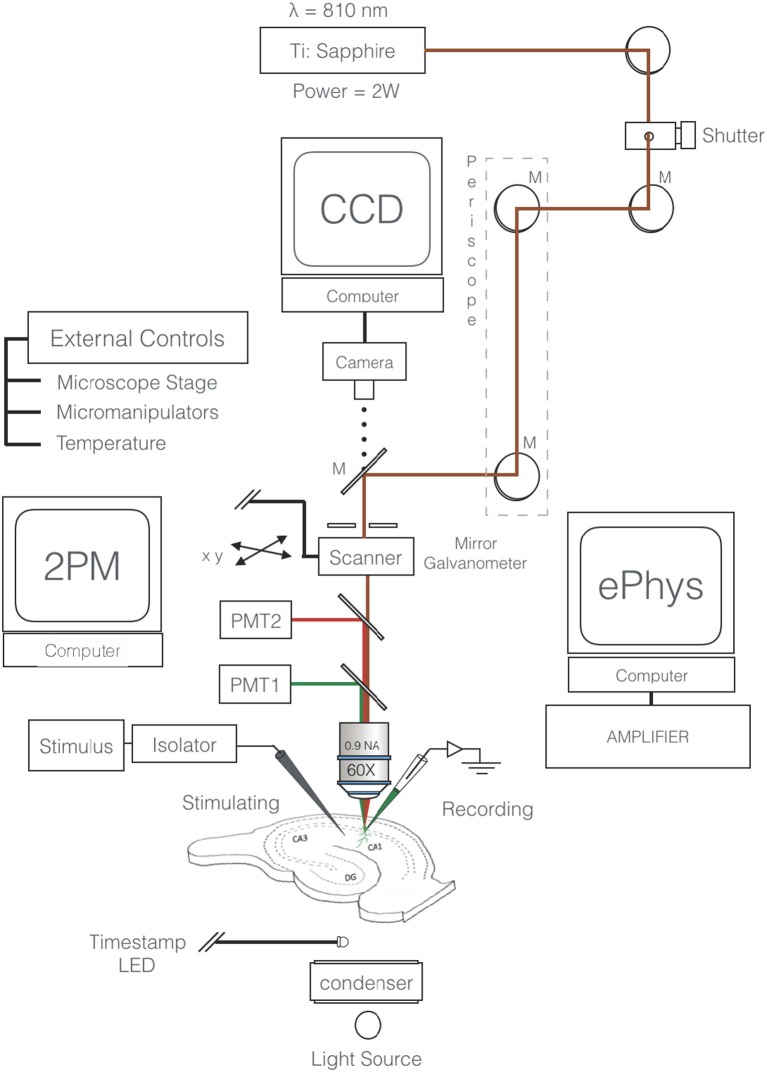
Schematic of two-photon excitation microscopy imaging and recording configuration. Excitation beam (red) is focused by a 60×, NA 0.9 objective to a diffraction limited spot that excites the fluorescent intracellular calcium indicator (e.g., Oregon Green 488 BAPTA-1). The target neuron’s membrane potential is constantly monitored through a somatic microelectrode. Excitation of inputs to the cell is achieved *via* a remote extracellular stimulating electrode (SE). Fluorescence is detected by a photomultiplier tube (PMT). A second fluorophore and secondary detector (PMT2 and dichroic) can be employed depending on the experiment. External control units for the micromanipulators, stage, and temperature are necessary components.

Slices are viewed through an upright microscope (e.g., Olympus BX51W1) equipped with a high numerical aperture water immersion objective (e.g., Olympus 60×, N.A. 0.9) *via* a confocal laser scan head (MRC1024MP, Bio-Rad Microsciences). Two-photon excitation is achieved using an ultrafast (100 fs pulses) Ti:Sapphire laser (Mai Tai, Spectra Physics: 3 W; 80 MHz). Emitted fluorescence is detected with a photomultiplier tube (PMT; H7422P-40 Hamamatsu) connected to a signal amplifier. If detection at an additional wavelength is required, a dichroic mirror is used to direct one waveband to a second PMT. Care should be taken when selecting fluorophores, to ensure that the emission spectra are non-overlapping. Two-photon excitation fluorescence images (“xy” and “xt” images) are acquired at 810 nm excitation and 15–20 mW average laser power in the focal plane, using LaserSharp software with 6× digital zoom. The microscope is also equipped with ordinary transmitted light and widefield fluorescence illuminators, digital camera, remotely controlled stage and micromanipulators, and temperature control units (Figure 1).

### Electrophysiological and Optical Recording

**Figure 2:**
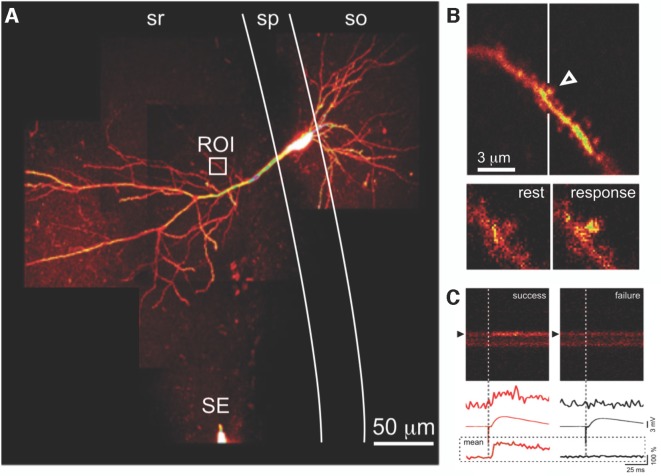
Optical detection of synaptic transmission. **(A)** CA1 pyramidal neuron, filled with fluorescent Ca^2+^ indicator. Presynaptic axons are activated by a SE in stratum radiatum (sr); evoked excitatory postsynaptic potentials (EPSPs) are recorded *via* a somatic microelectrode (not visible). Fluorescence changes due to calcium transients evoked by the same stimulus in an apical dendritic segment (region of interest indicated by the white box) are seen at higher magnification in **(B)**. **(B)** Evoked postsynaptic calcium transients (EPSCaTs) are restricted to an individual dendritic spine (arrowhead), seen below at higher magnification in video frames at rest (bottom left) and immediately after synaptic activation (bottom right). **(C)** EPSCaTs monitored *via* line-scan (x-t) imaging across the spine (black arrowhead) and adjacent dendritic shaft. Successful synaptic transmission (left), visible as a fluorescence increase, can be clearly distinguished from transmission failure (right). EPSPs during transmission failure at this synapse are due to successful transmission at some of the other synapses activated by the same extracellular stimulus. Traces show (top to bottom) single-trial fluorescence from the spine, averaged EPSP, and averaged fluorescence from the spine, during success (red, left) and failure (black, right). sp, stratum pyramidale; so, stratum oriens. Figure adapted from Enoki et al. (2009).

For electrophysiological recording, sharp microelectrodes minimize undesirable diffusion of cytoplasmic constituents out of, and micropipette solution into, the target neuron (Malinow and Tsien, 1990; Enoki and Fine, 2005). A disadvantage of sharp microelectrode recordings is that a small but persistent non-selective leak conductance may occur around the site of impalement; if patch-clamp recordings are required, perforated patch configuration (Lindau and Fernandez, 1986; Horn and Marty, 1988) is preferable, to minimize perturbation of the intracellular milieu. Selected pyramidal cells in the CA1 region of the hippocampus are impaled with sharp glass microelectrodes (80–120 MΩ) under widefield illumination and visual control *via* a digital camera. Microelectrodes are filled with a fluorescent Ca^2+^ probe (e.g., 0.5–1 mM Oregon Green 488 BAPTA-1 in H_2_O), optionally also with spectrally-distinct Ca^2+^-insensitive fluorophore (e.g., Alexa 594; Goldberg and Yuste, 2005) to serve as a morphological marker, and backfilled with 3 M KCl. Ionophoretic loading of cells is achieved by delivering low frequency (2 Hz) hyperpolarizing current pulses (~100–200 pA) *via* the intracellular amplifier (e.g., Multiclamp 700B, Molecular Devices, San Jose, CA, USA). After 5–20 min of loading, fluorescence in the soma and processes can be easily visualized (Figure 2A).

Dye loading of the target cell can be followed by two-photon excitation imaging using the lowest possible power. Once sufficient loading is achieved, hyperpolarizing pulses are discontinued; note that leakage from the pipette tip may contribute to additional loading over time. To assess the adequacy of loading, an action potential (AP) is evoked by depolarizing current injection, and corresponding fluorescent Ca^2+^ responses examined in the soma and proximal dendrites. As a useful guide, for adequate detection of EPSCaTs in dendritic spines, it should generally be the case that back-propagating APs cause a fractional change (%Δ*F/F*) >80% in Ca^2+^ probe fluorescence in the spines.

The extracellular stimulating electrode (SE), a sharpened, insulated, tungsten electrode (or theta-glass micropipette backfilled with 1 mM NaCl for minimal stimulation; Enoki et al., 2009), is placed in the stratum radiatum (sr) at distances not less than 50 μm (but <500 μm) from the soma, at a depth similar to the target dendrite and typically 50–200 μm from the border of the stratum pyramidale (Figure 2A). The extracellular stimulating pulses are increased to an intensity sufficient to elicit an AP-evoked Ca^2+^ transient in the soma and dendrites and then decreased by 50%–70% to a level at which subthreshold excitatory postsynaptic potentials (EPSPs) are reliably evoked.

### Optically Searching for EPSCaTs

Pairs or triplets of extracellular stimuli (each 100–300 μs square pulses of intensity described above) separated by 70 ms are delivered to the tissue preparation and maintained at a constant level throughout the searching procedure. Multiple stimuli are used to increase the likelihood of finding low *p_r_* synapses. The proximal region of the secondary and tertiary apical dendrites of the dye-filled CA1 pyramidal neuron is then systematically searched using fast raster scanning (e.g., 128 × 128 pixels), while simultaneously stimulating at a low frequency (~0.05–0.1 Hz), until a spine exhibiting an EPSCaT is located (Figure 2B). Low stimulation frequencies are maintained during the searching procedure to prevent unintended plasticity induction. When optically searching the dendritic branches it is important to follow a consistent strategy to avoid unintentionally neglecting or re-searching branches. A strategy widely used in our lab is the “wall follower” (right or left-hand rule). Given the remote positioning of the SE relative to the apical branches, the location of responsive spines and the time needed to find them can be highly variable; spines positioned proximally, however, tend to be more easily found than those at more distal locations. With this in mind, searching for responsive spines should take no longer than 45 min per cell, and if no responsive spine can be found within that time the cell is abandoned; another cell, far enough away to minimize overlap of its dendritic arbor with that of the previous cell, is impaled and filled, and the search for a responsive spine is repeated.

Once a responsive spine has been identified, line scanning (“xt” images, Figure 2C) can be used to image with better temporal resolution in order to record EPSCaTs with greater fidelity. Line scans ranging from 100 to 200 successive sweeps at 2 ms intervals are obtained along a line passing through the center of the activated spine (Figure 2B) and subjacent parent dendrite. It is important to minimize the duration and intensity of target irradiation to reduce phototoxicity and indicator bleaching. A scan rotator (Scientific Systems Design, Mississauga, ON, Canada) can be used to orient the scan trajectory, and an LED near the photodetector can be used to insert into the xt image a precise optical marker of onset of electrical stimulation (Figure 2C). The stimulating intensity is continually decreased until the threshold for EPSCaT detection is established; once established, the stimulating intensity is then incrementally increased for the experiment to a level (approximately 20% above this threshold) that minimizes the likelihood of stimulation failures of the afferent fibers (see “Conclusions and Perspective” section).

### Estimating Release Probability

We (Emptage et al., 1999) and others (Yuste and Denk, 1995; Yuste et al., 1999) have provided evidence that the probability of a presynaptic stimulus evoking an EPSCaT in a postsynaptic spine (*p_Ca_*) is equivalent to *p*_r_, the probability that the stimulus evoked transmitter release from the unlabeled, and thus invisible, presynaptic bouton. A useful estimate of *p*_r_ (a measure of the “reliability” of the synapse) can therefore be achieved by delivering a sufficient number of stimuli (~20–25 trials) to afferent fibers while recording EPSPs and EPSCaTs from the postsynaptic neuron. A failure method can be used, whereby *p*_r_ is related to the number of successes within a sample of trials assessed over a given period of time:

$$p_{r}=N_{\text{success}}/N_{\text{trials}}$$

where *N*_success_ is the number of successful transmission events over *N_trials_*, the total number of trials.

The Ca^2+^ transient amplitude is usefully expressed as

$$\text{\%Δ}F/F=100(F_{\text{transient}}-F_{\text{background}})/(F_{\text{initial}}-F_{\text{background}})$$

where *F*_initial_ is the mean fluorescence intensity of the imaged spine over a 20–40 ms time window prior to stimulation, *F*_transient_ is the mean fluorescence intensity after stimulation, and *F*_background_ is the mean intensity in regions devoid of labeled structures. To improve the signal-to-noise ratio, *F*_transient_ is measured over a 10–30 ms window encompassing the peak of the Ca^2+^ transient (Enoki et al., 2009). Using this approach, an event may be counted as a success if the EPSCaT amplitude exceeds the unstimulated noise amplitude, a threshold that is typically %Δ*F/F* >20%. Once sufficient recordings of EPSCaTs and EPSPs have been obtained, yielding a stable ratio of successes to failures, long-term synaptic plasticity may be induced using any of several available protocols. Importantly, we select spines with baseline *p*_r_ neither too high (<0.7) nor too low (>0.3) to avoid ceiling or floor effects that could mask the outcome of the chosen plasticity protocol.

### Modifications of Synaptic Efficacy

Various protocols can be used to induce long-term changes in synaptic efficacy. LTP may be induced using a spike-timing dependent plasticity (STDP; Song et al., 2000) protocol, wherein postsynaptic spiking is evoked shortly after a presynaptic stimulus (Markram et al., 1997; Bi and Poo, 1998; Nevian and Sakmann, 2006). Specifically, each EPSP is followed by (Δt = ~10–50 ms) the delivery of three pulses (at 100 Hz) of 2–10 ms postsynaptic depolarization (amplitude sufficient to evoke at least one AP), with 100 repetitions of this pairing at 0.33 Hz. LTD can also be induced with an STDP protocol, involving repetitive delivery of a postsynaptic AP preceding a single presynaptic stimulus (Feldman, 2012). Alternatively, a high-frequency stimulation (HFS) protocol may be used to induce LTP, where three bursts, at 1.5 s intervals, of 20 presynaptic pulses @ 100 Hz (with, if needed, sufficient simultaneous postsynaptic depolarization such that at least some of the presynaptic stimuli evoke APs (Emptage et al., 2003; Enoki et al., 2009); conversely, a low-frequency stimulation (LFS; e.g., 1 Hz) protocol may be used to induce LTD. It should be borne in mind that distinct mechanistic processes may result from different patterns of neuronal activity (Padamsey and Emptage, 2014).

### Re-evaluating Release Probability

Once the induction protocol is finished, *p_r_* can be reassessed at desired time points using procedures outlined above (see “Estimating Release Probability” section). Statistical comparisons between initial *p*_r_ and post-plasticity *p*_r_ are made off-line using appropriate statistics. Using these experimental procedures, our results have consistently indicated that long-term synaptic plasticity in non-silent synapses involves changes in *p*_r_. The precise molecular processes governing such changes and the contribution of altered modes of vesicular fusion (Choi et al., 2003) remain important unsettled questions even under these experimental circumstances. Furthermore, the presence of changes in *p*_r_ does not in itself establish the relative contribution of other possible mechanisms, such as alterations in quantal amplitude *q*, to changes in the compound EPSP amplitude.

### Estimating Synaptic Potency

**Figure 3:**
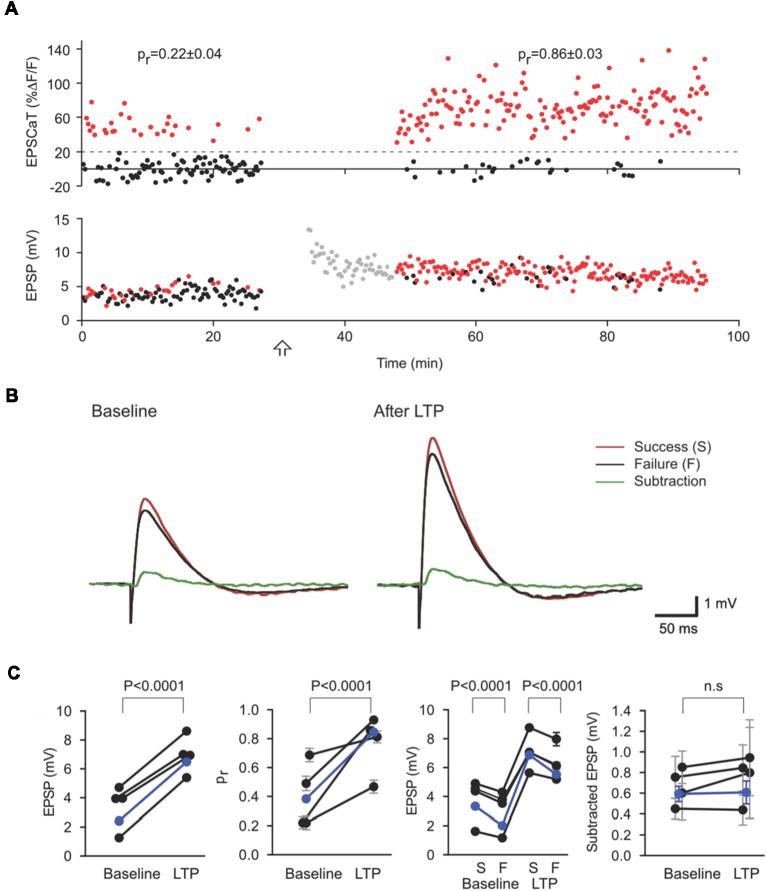
Subtractive analysis of unitary EPSP as an estimate of quantal size. **(A)** EPSCaT amplitudes (above) and EPSP amplitudes (below) recorded before and after long-term potentiation (LTP) induction. Corresponding EPSP and EPSCaT amplitudes are color-coded on the basis of EPSCaTs, with successes in red and failures in black. **(B)** Mean EPSP traces corresponding to EPSCaT successes (red) and failures (black). The difference between these averages (Subtraction, green) represents the mean contribution to the EPSP (i.e., the unitary EPSP) from the imaged active synapse. Traces shown are means before (Baseline; left) and 20–60 min after (right) LTP induction. LTP results in large increases in the overall mean EPSP and *pr* at the imaged synapse. The unitary EPSP amplitude from this imaged synapse, however, does not significantly change. **(C)** Values of compound EPSP, *pr*, EPSPs grouped according to success (S) or failure (F), and unitary EPSP amplitude from the imaged synapse. As revealed by such subtractive analysis, LTP induction in these experiments led to significant and corresponding increases in *pr* at the imaged synapse and in the (multi-synaptic) EPSP, with no significant change in the unitary EPSP from the imaged synapse. Figure adapted from Enoki et al. (2009).

Electrical recording by itself has proven inadequate to resolve unambiguously the magnitude of the evoked response from an individual synapse (sometimes called the “potency” of the synapse) that contributes to a compound EPSP. Conjoint EPSCaT recording, however, permits a subtractive analysis that can effectively address the ambiguity. On average, compound EPSP amplitudes are larger in trials where the imaged synapse releases transmitter than in those where the imaged synapse fails; indeed, subtracting the mean EPSP in failure trials from the mean EPSP in successes yields an estimate of the mean unitary EPSP from the EPSCaT-generating synapse (Figures 3A,B):

$${\bar{\text{EPSP}}}_{\text{success}}-{\bar{\text{EPSP}}}_{\text{failure}}={\bar{\text{EPSP}}}_{\text{unitary}}$$

With adequate sample sizes, this procedure can provide a reliable estimate of the mean unitary amplitude of the evoked response at the imaged synapse, and its potential modifications. Using this subtractive analysis, we have demonstrated that LTP at mature CA3-CA1 synapses is associated with increases in synaptic reliability (i.e., in* p_r_*) while changes in potency (i.e., in ${\bar{\text{EPSP}}}_{\text{unitary}}$) are negligible (Enoki et al., 2009; Figure 3C).

### Optically Confirmed Minimal Stimulation

**Figure 4:**
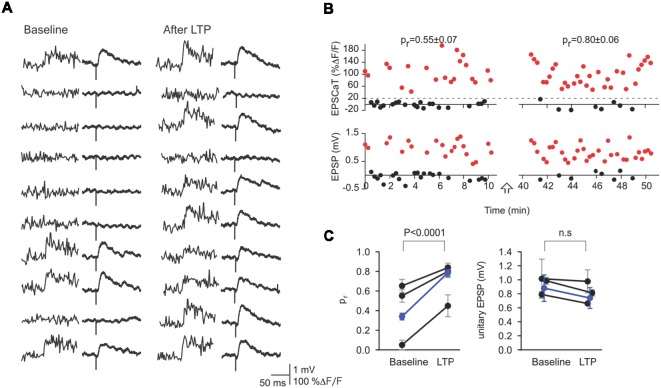
Minimal stimulation and optical quantal analysis. **(A)** Representative sequential traces showing the perfect correspondence between success or failure of EPSCaTs (left) and EPSPs (right) before (Baseline) and After LTP. This constant correspondence provides strong evidence that the stimulus in this experiment activated only the imaged synapse and that EPSCaTs are reliable reporters of transmitter release. **(B)** EPSCaT (above) and EPSP amplitudes (below) recorded from this synapse before and after LTP induction. LTP induction increased *pr* but not the unitary EPSP amplitude. **(C)** Values of *pr* (left) and unitary EPSP (right) from the imaged synapse for this and two other experiments (black) are shown before and after LTP (weighted means shown in blue). Such optically confirmed minimal stimulation demonstrates that LTP induction leads to significant increases in *pr*, with no significant change in unitary EPSP amplitude. Figure adapted from Enoki et al. (2009).

The above conclusion is supported by other methods also enabled by conjoint optical and electrical recording. As noted previously, minimal presynaptic axon stimulation procedures (Raastad, 1995) suffer from ambiguity as to the actual number of synapses activated (Dobrunz and Stevens, 1997), as even single CA3 axons may make multiple contacts with a single CA1 neuron (Sorra and Harris, 1993). By combining optical quantal analysis with minimal presynaptic stimulation, however, such ambiguity can be eliminated, permitting a direct comparison of the contributions of *p_r_* and unitary EPSP amplitude: in those cases where only the imaged synapse is being activated, there will be perfect correspondence between EPSCaTs and EPSPs for both successes and failures (Figures 4A,B). In all such cases, LTP-inducing stimuli increased *p*_r_ but had no effect on the amplitude of unitary EPSPs (Figure 4C; Enoki et al., 2009). These optical quantal analyses provide strong evidence that LTP at CA3-CA1 synapses is expressed chiefly through an increase in synaptic reliability, i.e., through an increase in *p*_r_. We note, however, that these experiments have been mainly restricted to synapses on proximal dendrites, and to effects on transmission at low frequencies, so that the generality of these results, even for this class of synapse, remains to be established.

## Conclusions and Perspective

Here we have outlined the procedures necessary to carry out optical quantal analyses at individual synapses within hippocampal slice preparations, and have summarized results on the mode of expression of LTP obtained by these methods. Despite the distinct advantages of optical over traditional electrophysiological quantal analyses, several items must be kept in mind in interpreting such experiments. Buffering of intracellular Ca^2+^ by Ca^2+^ indicators could in principle interfere with calcium-dependent postsynaptic aspects of LTP expression, though this is unlikely given that the magnitude of LTP is unaltered by indicator loading (Enoki et al., 2009). Selection of spines for analysis may exclude small spines beyond the limit of optical resolution, or spines with small EPSCaTs (e.g., less mature spines lacking endoplasmic reticulum; Spacek and Harris, 1997). Observed spines, however, appear to account for the majority of the evoked response (Enoki et al., 2009). Additionally, although our extracellular stimulation protocols reliably induced APs, we have not excluded the possibility that some EPSCaT failures reflect factors other than *p_r_*, e.g., failure of APs to reach the terminal, or stochasticity of Ca^2+^ store release, though this seems unlikely given that the probability of evoking EPSCaTs is influenced by the same factors that influence *p*_r_. Thus, notwithstanding the experimental constraints that limit trial numbers and thus the precision of *p*_r_ determination, our estimates of *p*_r_ using the procedures described here have been reliably and predictably influenced by manipulations known to alter vesicular release (Emptage et al., 1999; Reid et al., 2001). Moreover, the fact that increasing stimulus intensity does not alter our estimate of *p*_r_ (Emptage et al., 1999) provides a compelling argument against the spurious effects of axon excitability.

Controversies remain regarding possible roles of changes in the number of transmitter release sites (Walmsley et al., 1987) and alteration in the amount of transmitter released per quantum (Choi et al., 2003; Midorikawa and Sakaba, 2017) in the expression of LTP and LTD (see MacDougall and Fine, 2014) for a unified model and more extensive discussion). Unfortunately, because CICR from internal stores contributes significantly and nonlinearly to the EPSCaT (Emptage et al., 1999), fluctuations in EPSCaT amplitude cannot resolve these controversies.

Although we have described this technique specifically in area CA1 of the hippocampus, optical quantal analysis can be carried out at other synapses (Reid et al., 2004; Chalifoux and Carter, 2010) and in other preparations (Sinnen et al., 2016) and model organisms (Newman et al., 2017). Importantly, this method can be adapted for *in vivo* investigations and functional mapping of cortical (Svoboda et al., 1997; Chen et al., 2011; Wilson et al., 2016; Scholl et al., 2017) and subcortical tissue, including the hippocampus (Mizrahi et al., 2004; Gu et al., 2014). Such applications have been facilitated by ongoing improvements in the useful depth of multiphoton excitation fluorescence microscopy (Theer et al., 2003; Kobat et al., 2009, 2011; Horton et al., 2013), adaptive micro-optics (Andermann et al., 2013; Velasco and Levene, 2014), genetically encoded voltage, Ca^2+^, and other optogenetic sensors (Akerboom et al., 2013; Storace et al., 2016; Yang and St-Pierre, 2016) and two-photon microendoscopy (Jung and Schnitzer, 2003; Bocarsly et al., 2015; Sato et al., 2017; Ohayon et al., 2018). At the same time, rapidly advancing developments in optical sensors for the detection of neurotransmitters, including but not limited to glutamate (Marvin et al., 2013; Helassa et al., 2018) and GABA (Masharina et al., 2012), provide exciting complementary strategies for optical quantal analyses both in organized tissue preparations (Borghuis et al., 2013; Jensen et al., 2017) and *in vivo* applications (Helassa et al., 2018). We expect that the wide applicability and power of optical quantal analysis will lead to its increasing use to reveal the mechanisms of synaptic transmission and their modifications in learning and other phenomena.

## Author Contributions

Both authors wrote the manuscript together.

## Conflict of Interest Statement

The authors declare that the research was conducted in the absence of any commercial or financial relationships that could be construed as a potential conflict of interest.
